# Comparative Evaluation of Reproducibility of Phage-Displayed Peptide Selections and NGS Data, through High-Fidelity Mapping of Massive Peptide Repertoires

**DOI:** 10.3390/ijms24021594

**Published:** 2023-01-13

**Authors:** Klaus G. Petry, Eleftherios Pilalis, Aristotelis Chatziioannou

**Affiliations:** 1 INSERM U1029 Tumor Microenvironment and INSERM U1049 Neuroinflammation, Bordeaux University, 33000 Bordeaux, France; 2 CNRS UMR5536 Centre Résonance Magnétique des Systèmes Biologiques, Brain Molecular Imaging Team, Bordeaux University, 33000 Bordeaux, France; 3 Enios Applications P.C., Kallithea, 17671 Athens, Greece; 4 Center of Systems Biology, Biomedical Research Foundation of the Academy of Athens, 11527 Athens, Greece

**Keywords:** phage display, bio-panning, NGS, peptides, protein domains, bioinformatics

## Abstract

Phage-displayed peptide selections generate complex repertoires of several hundred thousand peptides as revealed by next-generation sequencing (NGS). In repeated peptide selections, however, even in identical experimental in vitro conditions, only a very small number of common peptides are found. The repertoire complexities are evidence of the difficulty of distinguishing between effective selections of specific peptide binders to exposed targets and the potential high background noise. Such investigation is even more relevant when considering the plethora of in vivo expressed targets on cells, in organs or in the entire organism to define targeting peptide agents. In the present study, we compare the published NGS data of three peptide repertoires that were obtained by phage display under identical experimental in vitro conditions. By applying the recently developed tool PepSimili we evaluate the calculated similarities of the individual peptides from each of these three repertoires and perform their mappings on the human proteome. The peptide-to-peptide mappings reveal high similarities among the three repertoires, confirming the desired reproducibility of phage-displayed peptide selections.

## 1. Introduction

Various screening strategies have been generated to identify biomarkers for disease development. Among them, the screening of phage-displayed peptide ligands, first described by Smith et al. [1] responds to the challenge of defining peptide biomarkers presenting interactions of high affinity and specificity towards a wide spectrum of targets.

The complexity of recombinant bacteriophages, expressing combinatorial DNA libraries at the 10^9^ order of variants, displays the equivalent complexity of peptides as fusion proteins on their surface. By using such combinatorial phage DNA libraries, the screenings performed allowed the cloning of peptides, both in vitro to a single macromolecule [2,3] and in vivo for discriminative vascular mapping [4] by organ-specific homing peptides [5], for targeting organelles in cells by internalizing homing peptides [6] in diffuse pathological situations, e.g., tumor development [7] and in experimental animal studies of neurodegenerative diseases [8,9,10].

For a few of the identified peptide ligands their interacting molecular targets could be discovered [11,12] allowing perspectives of molecular targeted imaging and selective delivery of therapeutics [13,14]. Phage-displayed peptide screenings performed in vivo or on cell cultures face the plethora of in vivo expressed target molecules and, consequently, generate the recovery of complex repertoires of binding peptides. Next-generation sequencing (NGS) technologies have shown the high complexity of isolated peptide repertoires [15]. Despite the development of software solutions for the NGS analysis of the comparable complexity of generated peptide repertoires [16] the limited choice of targeting peptides was mainly caused by either the enrichment of the main consensus peptide sequences during the selection procedures, or the particular presence of a number of peptides when compared to control selections. This is further supported by physical subtraction experiments of comparable target vs. control selected phage repertoires [8].

Based on the strategies as suggested in the experimental procedure manuals of phage-expressed peptide library selections (for review [17]), most of the investigations have focused on cloning out a single peptide or a small number of peptides as tools for further development and study, such as histopathology characterization, in vivo imaging, targeted therapeutic vectorization or vaccine development (for review [18]).

Furthermore, the analysis of retained complex peptide repertoires evidenced the difficulty of distinguishing between effective selections of specific peptide binders to exposed targets and the potential high background noise. Indeed, NGS analysis of repeated peptide selections performed under the same conditions, even when performed in vitro against a single cell line, that have generated large repertoires of unique peptides, showed that only a very small number of peptides are present in the comparable selections [19]. The observation, however, that the vast majority of retained peptides—representing several hundreds of thousands of peptides in each of the experimentally identical selections—are different, calls into question the efficient selectivity of phage-displayed peptide selections. Such inquiry is even more relevant when considering the plethora of in vivo expressed targets on cells, in organs or in the entire organism. The chosen limitation of a few retained peptides, alongside inclusion of some technical biases, suggests that the retained repertoires might represent, across their vast number, random peptides of which only a very few can be considered as effectively selected for molecular and cellular targets [20].

Based on observations of peptide selections and the resulting NGS data we assume that a large number of the vast spectrum of peptides could be perfectly selected against the exposed molecular targets.

Such consideration, especially in the case of in vivo targets, is even more convincing when peptide selections were performed under identical experimental conditions, and reveal, however, only a relatively small number of common peptides within the generated comparable repertoires.

Assuming the potentially effective selection of the large spectrum of peptides from the combinatorial expression library, it is emphasized that the in vivo selected peptides would greatly mimic proteins, particularly the segments of functional epitopes/domains being involved in physiological interaction with exposed target molecules. Such mimicking of a functional protein domain would retain at least several peptides in the selected repertoire, but with a high degree of similarity of linear amino acid sequences. Consequently, generated repertoires would contain peptides demonstrating perfect likeness to a protein domain and peptides of strong similarity. It is important to mention that the high similarity of retained peptides to the same domain is measurable. Furthermore, repertoires generated under comparable conditions would contain a large spectrum of peptides with measurable similarity to each other.

In the present study, we challenge our hypothesis by studying the published data of three peptide repertoires that were generated under identical experimental conditions by Brinton et al. [19]. These three repertoires contain only a relatively small number of common peptides among the several hundreds of thousands of retained peptides in each selection.

To analyze the three generated peptide repertoires that were obtained by in vivo biopanning phage display with a combinatorial library of cyclic 7-mers peptides on a cancer-associated fibroblast (CAF) cell line [19], we used a new approach. By applying the recently developed computational galaxy pipeline PepSimili [21] we evaluated the calculated similarities of the individual peptides from each one of the three repertoires to the peptides of the others and perform for all three peptide repertoires their mappings on the human proteome. The calculated mapping scores allowed a comparative ranking of proteins. Among the first 200 ranked proteins of these three mappings, we compared several examples of the peptide mappings from the three selections, revealing putative protein domains/epitopes that are mimicked by the selected peptides. Overall, the PepSimili application demonstrated that the three individual peptide repertoires generated against the CAF cell line show very high similarities, confirming the desired reproducibility of phage-displayed peptide selections. To our knowledge, this is the first objective study to compare massive peptide repertoires obtained by high throughput sequencing of phage display libraries to evaluate the data reproducibility in the generation of massive peptide selections.

**Figure 1:**
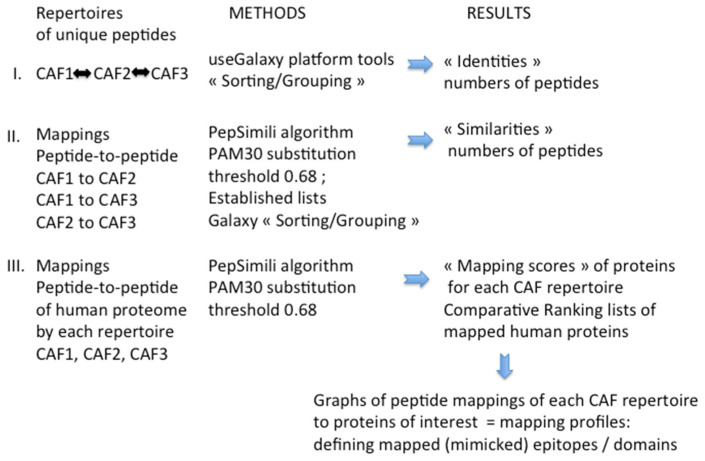
Overview of experimental design. The bioinformatics study of the three CAF peptide repertoires was performed in a stepwise approach. The main steps are indicated by I/II/III. The various methods applied that led to the generated results are summarized.

An overview of the bioinformatics study is presented in Figure 1.

## 2. Results

### 2.1. Confirmation of Data Analysis of the Three CAF Peptide Selections

**Figure 2:**
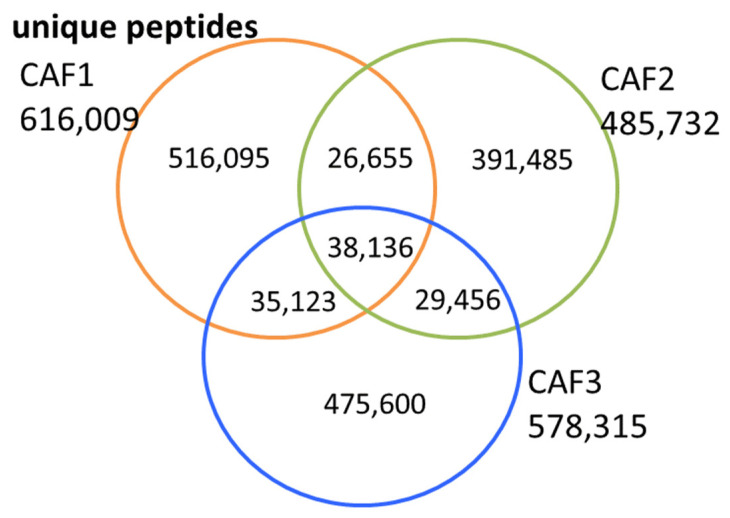
Venn diagram of three peptide selections on CAF cell line. Total numbers of unique peptide sequences of the NGS data derived from individual translated DNA sequences are given for each of the three CAF repertoires. The numbers inscribed within the circles reflect the unique sequences for each repertoire and the numbers of peptides in overlapping segments being observed, respectively, in two or three of the peptide selections.

Based on the published data of the three phage-displayed 7aa peptide repertoires that were selected in vitro against the CAF cell line [19] we performed a qualitative analysis to compare the three repertoires with each other. As the authors have provided all the deep sequencing data of their very complex study, it was possible to work on the obtained peptide repertoires. While considering all peptides of the three repertoires as unique peptides, independently of their occurrence in the respective selections, we confirm their observations as shown in the Venn diagram (Figure 2). Indeed, among the peptides of the three CAF repertoires only small numbers of peptides are common to two or three of the three distinct CAF repertoires. Only 4% and 6%, respectively, of the CAF1 peptide repertoires were identical to CAF2 and CAF3 repertoires, and 5% were identical between CAF2 and CAF3 repertoires.

As most of the peptides within the individual repertoires (>94%) were not common to the various in vitro selections towards a single cell line, even though the studies were performed under the same experimental conditions, it could be argued that selections of a few “specific” peptides are obtained by chance while the very large amount of the retained peptide repertoires is potentially background noise.

### 2.2. Similarity Evaluation of Peptides among the Three CAF Repertoires

With the hypothesis that the peptides being retained in the three CAF peptide repertoires could present certain degrees of similarity in order to be positively selected, we performed a similarity evaluation of peptides. We applied the PepSimili algorithm [17] to integrate the similarity of the selected peptides to each other (CAF1 to CAF2, CAF1 to CAF3, CAF2 to CAF3). The PepSimili algorithm is based on the peptide-to-peptide mapping and the similarity score calculation including the PAM30 substitution. By applying for 7 aa peptides the strong threshold of 0.68 calculation (maximum 1), 57% and 66% respectively of the peptides of the CAF1 repertoire present strong similarity to those of the CAF2 and CAF3 peptide repertoires, and 61% of peptides present strong similarity between CAF2 and CAF3 peptide repertoires (Table 1).

### 2.3. Similarity Evaluation of Selected CAF Peptides with Human Proteins

**Figure 3:**
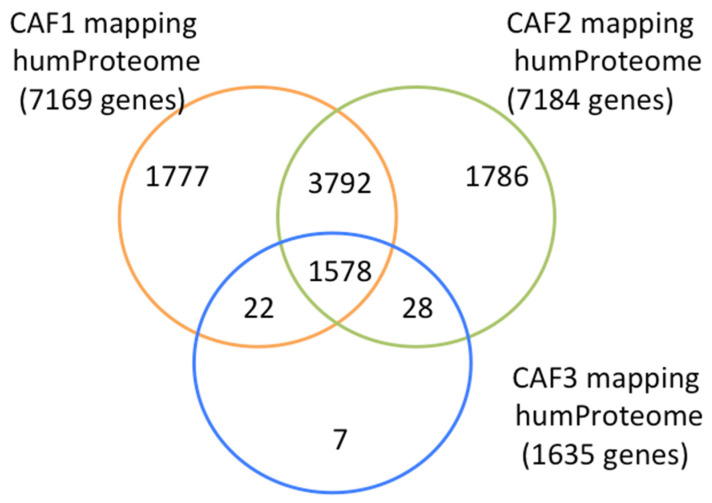
Venn diagram presentation of mappings of three CAF peptide repertoires on human proteome at threshold 0.68. The numbers of mappings for the retained proteins by three individual CAF peptide repertoires, the over-lapping of mapped proteins and the individually mapped proteins are indicated by inscribed numbers.

We performed the similarity evaluation of the three individual peptide repertoires to the human “personalized” proteome of 20,000 proteins/genes (20 K) by peptide-to-peptide mappings, which is an integrated application of the PepSimili bioinformatics modules. Again, applying the PAM30 threshold score at 0.68, the peptides of the CAF1 repertoire were mapped to proteins encoded by 7169 proteins/genes, the peptides of the CAF2 repertoire to 7184 proteins/genes, and the peptides of the CAF3 repertoire to 1635 proteins/genes. Among these evaluations, 1578 proteins/genes were mapped by all the three CAF peptide repertoires, while the same 5370 proteins/genes were mapped by both CAF1 and CAF2 repertoires, 1600 proteins/genes were mapped by both repertoires CAF1 and CAF3 and 1606 proteins/genes were mapped by both CAF2 and CAF3 repertoires. The data are summarized in the Venn diagram (Figure 3).

Based on these peptide-to-peptide mappings of the three CAF peptide repertoires with the human proteome we considered the established PAM30 scores for the ranking of the proteins mapped by the selected peptides. The ranking lists of proteins mapped by the three individual CAF peptide repertoires are presented in Supplementary File S2 (Table lists_signMapp_hprots_by_3clCAFs). Lists of proteins with the highest rankings for mappings of the three individual CAF repertoires are presented in Table 2. Many proteins present similar high rank positions.

In order to define a robust significant parameter in the ranking of mapped proteins, we also performed the mapping of all three peptide repertoires (CAFtotal, unique peptides), considering them as a common indicator for mimicking. Among the mapped proteins by peptides of CAFtotal, the cut-off line was calculated at protein in rank position N°1926 (*p* = 0.01 significant).

**Figure 4:**
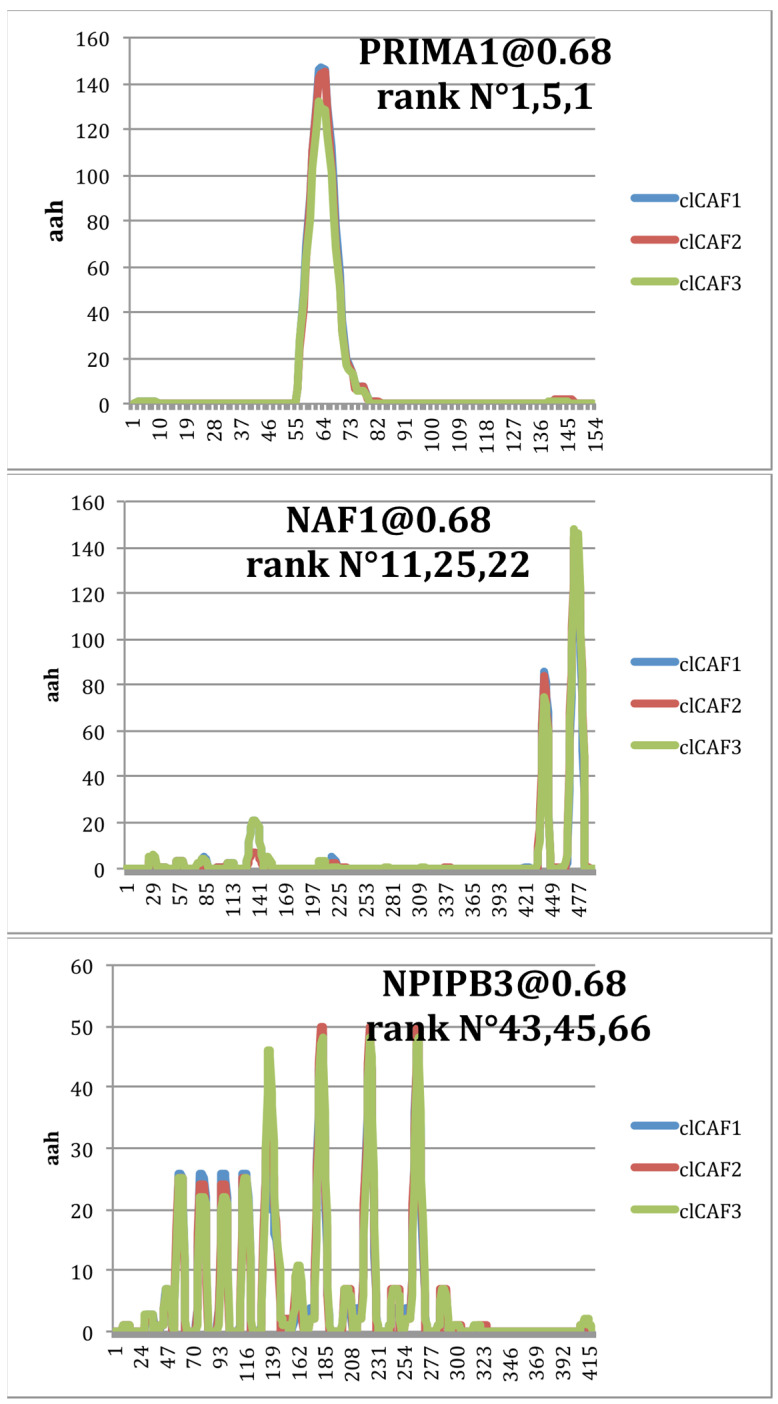
Mapping profiles of three CAF peptide selections on human proteins. The mapping profiles (aah) on three examples of proteins show very high similarity of the three CAF peptides selections (indicated by different colors). Score rank N° of the protein is indicated for CAF1, CAF2 and CAF3, respectively. For simplified visualization, the presented examples were chosen according to the length of proteins (<500 aa).

We then used the generated individual peptide-to-peptide mappings on the proteins of the human proteome for the visualization of the mapping profiles obtained by the three selected peptide repertoires. Based on the ranking score referred to in Supplementary File S2 (Table lists_signMapp_hprots_by_3clCAFs), three examples of peptide mappings on proteins ranked among the first 70 are shown (Figure 4). The peptide mappings to human proteins by each of the three CAF peptide repertoires present very similar profiles of protein segments. The similarity slightly decreases for the lower-ranked proteins (not shown). Further examples of aah (aa mapping hits) and the corresponding protein sequences are presented in Supplementary File S1 (Figure CAFs_hprot@0.68_profiles 20220407kgp).

## 3. Discussion

To address the question of effectively selected peptides or the randomness of positive selected peptides among selections of phage-displayed peptide repertoires, we used the data published by Brinton et al. [19]. In their study, the authors present data of the comparative selections of peptide ligands by in vitro biopanning phage display with a combinatorial library of cyclic 7-mer peptides on a cancer-associated fibroblast (CAF) cell line. The in vitro selections were performed in three independent experiments under identical conditions (same cell line, same culture conditions, same recombinant phage library, etc). The three selections were sequenced by NGS revealing three large peptide repertoires, which are each composed of several hundreds of thousands of unique peptides.

The authors had already observed that the three repertoires CAF1, CAF2 and CAF3 contained only a relatively small number of peptides that were common to the three selections. Indeed, the common fraction of peptides of the three repertoires represents about 5% of the selected peptides. By applying their developed PHASTpep software [19], the authors identified three potential CAF-selective peptide sequences as present in these three CAF peptides repertoires, but absent from peptide selections performed with other cell lines and under various other experimental conditions serving as controls. Finally, their study confirmed experimentally for two of these three identified peptides the binding to the tumor microenvironment in vitro and in vivo. This experimental observation is important to notice as indicative evidence that the selections were effectively producing valuable peptide ligands. On the other hand, the several hundreds of thousands of non-common peptides of the three CAF repertoires were not further studied. Among them, some were observed in the various control selections. Furthermore, these large peptide fractions could potentially include peptides that were generated by the biased production of false binders, which is commonly considered to be background noise. Overall, they were considered as non-relevant peptides in specific binding to the CAF cell line during the phage display selection process. The fact of excluding several hundreds of thousands of peptides from comparable selections, however, raises major doubts concerning the effective selection of peptides within the randomness of retained phage-displayed peptide selection repertoires, even when selections were performed in vitro under identical experimentation.

The present study confirmed that only a small number of peptides (about 5% of the total peptide repertoires) is common to the three generated CAF repertoires. The data analysis of the three peptide repertoires, however, does not consider that peptides presenting a different, albeit minor similarity in the amino acid sequences, could have the same biological function in binding to molecular targets that are expressed by the CAF cell line.

Based on the provided NGS peptide repertoires, we performed an analysis by using the PepSimili algorithm [21]. The tool performs peptide-to-peptide mapping of massive peptide repertoires obtained by high-throughput sequencing of phage display libraries to the proteome. For this, each protein is split into sequential peptides of the length that corresponds to the object of the study, in the present case 7 aa. The peptide-to-peptide mapping integrates the PAM30 substitution matrix for the calculation of similarity, whereby the chosen high threshold provides a parameter for retaining peptides of significant similarity, and generating their ranking. It also allows consideration of each of the three CAF repertoires as sequential peptides for mapping and comparative evaluation.

By this analysis we observed, first, the application of the peptide-to-peptide mapping of the PepSimili algorithm reveals that the peptides among the three CAF repertoires present a high degree of similarity, which integrates about 60% (57% to 66%) of the three CAF peptide selections. Second, the peptide-to-peptide mapping of the CAF peptides to human proteome reveals that the selected CAF peptides present high similarities with linear fragments of human proteins. The extent of such mappings (mapping score) allows ranking of the mapped proteins for further functional and interactive analysis such as BioInfoMiner.

The observed similarity of peptides within the repertoires and among the three repertoires could be based on diverse DNA sequences of the recombinant library encoding enriched identical peptides and also to a certain degree on those encoding peptides presenting the calculated similarity. We did not study such diversity at the DNA encoding level as we considered that the effective peptide selection acts on the amino acid sequence and the imposed interactive structure in biopanning, rather than on the genotypes. We do not, however, exclude that the potential diversity in DNA sequences encoding enriched identical peptides and those of strong similarity, could be a strong indication of phenotypic vs. random selection. In our study, we worked with unique peptides without discrimination of their occurrences in the selections. Considering peptides exclusively encoded by unique DNA sequences, on the other hand, could point to genetic bottlenecks. In our study, we did not integrate the genotypic diversity of the retained peptides.

Considering the similarity between the individual peptides among the several hundreds of thousands of peptides for each of the three CAF repertoires on whole proteome, the mapping of protein fragments by the peptides could serve as indicators for the potentially mimicked putative domains/epitopes of proteins in their interaction with the molecular targets that are expressed by the CAF cell line. The identification of more than one putative domain/epitope within an identified gene/protein could further reflect the complex structural interaction of the mimicked protein with the target molecule.

Indeed, the PepSimili analysis reveals that the three independently in vitro selected phage-peptide repertoires against the CAF cell line have a very high output of mimicking proteins that are potentially involved in active binding with CAFs. The ranking of the peptide-to-peptide mapping scores reveals that most of the proteins among the first 200 listed of each of the three peptide selections present major convergence in ranking position. The similar mapping profiles that are generated by the three CAF repertoires for the most prominent human proteins, covering the same linear amino acid sequences of protein fragments, is furthermore an argument for the strong data reproducibility of the three peptide selections towards the CAF cell line.

The present study provides an overview of similarities between peptides from three CAF selections and their common mappings of human proteins. It is not the scope of the present study to define CAF-specific peptide ligands, nor the specificity of human protein mappings to specifically CAF expressed targets. Such specificity could be established when comparing the data of CAF selections with repertoires from other selections. Indeed, the CAF repertoires could well contain peptides that are binding to other cells and consequently the defined mappings of protein domains/epitopes could interact with other cell types.

To our knowledge, this is the first comparative evaluation of several independently generated peptide repertoires confirming the strong data reproducibility of the selections of massive peptide repertoires by the unbiased similarity calculation, using a bioinformatics tool. The unbiased detection of putative protein domains/epitopes, that could serve as indicator for the reproducibility of screening selections, is obtained by the effective mimicking with selected peptides. Even if only considering the mimicked protein domains/epitopes of the human proteome, which serves as input to the present study, the PepSimili approach reduces the background noise that is considered typical within NGS data of phage display selections.

Among the remaining considered potential background of peptides, some of them might, however, mimic other biofunctional structures, like carbohydrates, that are interacting with the cellular targets. Such potential non-protein mimics by peptides cannot be identified in the PepSimili tool application currently, but could be integrated in a future version.

Defining the output of mimicked proteins by peptide repertoires is the objective of many bioinformatics tools that have been developed in the past years for the analysis of NGS phage-displayed peptide repertoires. Indeed, based on NGS peptide repertoires, such tools define relevant peptide ligands and predict epitopes (for review [22]) and allow computational prediction of epitope-structural characteristics, mechanisms of action and potential biological activity (for review [23]).

In line with defining a whole spectrum of potentially functional protein domains is the development of applications of the web tool InteractomeSeq [24]. The first particular application that is related to the definition of the domainome of *Helicobacter pylori* opens the identification of new potential biomarkers for the infectious agent and further innovative developments of biomarker profiling, reverse vaccinology and structural/functional studies.

The PepSimili algorithm as shown in its present application to the CAF study allows the comparative analysis of several large peptide repertoires. Viewed in perspective, the tool provides major advantages. Methodologically, while NGS allows the enrichment evaluation of peptide binders to targets during rounds of the selection-amplification process, the PepSimili approach could find application to comparatively evaluate NGS peptide selections in repeated first-round experiments to targets performed in parallel and in addition to appropriate controls. Such a combined approach would avoid the enrichment biases in amplification rounds. The comparative mappings of target selections vs. appropriate controls, which is an integrated part of the PepSimili workflow, would help to define mimicked functional protein domains/epitopes.

The mapping of the whole proteome by the PepSimili tool could be further extended to comparative studies of selections of peptides that were generated with recombinant phage libraries encoding different sizes of peptides performed in different laboratories. Such a comparison could help to evaluate the efficacy in defining protein domains/epitopes as interactive ligands towards a very large spectrum of molecular targets in biological studies both in vitro and in vivo. The identification of protein domains/epitopes by mimicking peptides from various selections with different recombinant libraries that are common in different selections will help to confirm the determination of the interacting protein fragments with the target cells. Alternatively, it could serve to distinguish between peptide repertoires generated in various diseases, leading to the confirmation of specific biomarkers in distinct diseases.

Although peptide mappings are mainly performed with the human proteome or those of mammalian species, potentially, mappings of generated peptide repertoires to proteomes other than mammals (i.e., non-mammalian vertebrates or non-vertebrates) could be envisaged to help in defining common functional domains. In this case, however, we underline the limitation that the PepSimili algorithm integrates the PAM30 substitution matrix and compares short peptides of a given number of amino acids to peptides of the same size in a linear way. PepSimili will be limited in defining and comparatively evaluating the functional capacity of a protein domain from an evolutionarily distant species (vertebrates and non-vertebrates) presenting the same structural formation and positive interaction side of an epitope, but which is based on a different amino acid sequence. Such investigations would need the integration of other evaluated substitution matrices. Indeed, we recall the understanding that for biological systems the spatial structure is more conservative than the protein sequence [25].

The synthesis of identified short linear protein fragments into interactive protein domains/epitopes could be further developed as in vivo targeting agents and in therapeutic applications. These protein fragments could serve as antigens for the generation of recombinant antibodies and small antibody fragments. The application of such biotechnological tools could provide potential competitive blocking agents for the molecular interaction of the mimicked protein domains/epitopes with their cellular receptors in biological systems.

## 4. Materials and Methods

For the comparative evaluation of the three peptide repertoires that were generated on the CAF cell line study [15], we applied the elements of the PepSimili workflow [21] that is implemented as a tool in a Galaxy cloud platform [26], online available. The bioinformatics modules of the Galaxy platform allow manipulation of the raw fastq files including quality filtering, trimming of the sequences to isolate the variable part of the recombinant phages and DNA-to-protein translation. To obtain detailed information on the common and different compounds among the three NGS peptide selections, named CAF1, CAF2 and CAF3, the peptide lists were filtered and sorted for duplicates to obtain unique peptides in each of the lists by using the corresponding tools of the Galaxy platform. These three lists of unique peptides were used as basis for further analysis (lists can be obtained open request). Further processing of data included the grouping or subtraction and sorting in demand of steps of the qualitative and quantitative analysis. All these applied tools were used online on the Galaxy platform [26].

We first performed a comparison of the individual peptides of the three peptide lists to quantitate the number of peptides presenting the sequence identity among the three selections.

In a second step, we performed the mapping of the peptides from each list to the peptides of the two others by using the module peptide-to-peptide mapping of PepSimili algorithm. The similarity between two peptides is calculated based on the implemented the PAM30 substitution matrix [27]. The algorithm defines significant peptide-to-peptide mappings for 7aa at thresholds ranging from 0.4 and 0.8 (maximum 1). We choose threshold 0.68 for mappings.

This approach allows testing of every single peptide of one repertoire to every single peptide of another peptide repertoire (for simplification this was done in a direct comparison of two repertoires at a time; CAF1 to CAF2, CAF1 to CAF3 and CAF2 to CAF3) The resulting lists of threshold criteria-fulfilling mappings were sorted. The presence of the peptides presenting “similarity” at threshold 0.68 among the relevant lists is counted and the similarity above the defined threshold for each comparison is expressed as the percentage of the respectively compared selected peptides.

Applying once more the PepSimili algorithm at threshold 0.68, we then performed peptide-to-peptide mappings using each set of peptides from the three CAF repertoires to the human proteome, here defined as 20 K (File hproteome20Kkgp.fasta; can be obtained upon request), which allows limitation to the most relevant interactive binding proteins in order to gain mapping computer time. For these mappings, the algorithm transforms the proteome in a set of peptides of 7aa. All the peptide mappings on each protein, which is based on the defined similarity PAM30 threshold of the peptide with the linear underlying protein segment, is indicated at the position of the mapping. The mapping module also integrates the generation of a random Mock repertoire. Mappings on the proteome with the Mock repertoire are automatically subtracted from the relevant mapping result of test repertoire. As there is no equivalent control repertoire to the CAF cell line, such potential mapping and subtraction is not possible in this case study. Altogether, the valuable mappings result in a profile of protein segments that are retained by the criteria-fulfilling peptide mappings. Thereby, the profile starts at the identified amino acid sequence with the change of the number of peptide mappings (hits) from 0 to =/>1 and ends at the protein sequence with the change from =/>1 to 0 hits. The covered segment(s) are retained for the calculation of the m(apping)-score, thereby being the sum of the mappings divided by the portion (numbers of aa) of the protein covered by them.

The mapping-score allows the ranking of the proteins mapped by the peptides of the individual selection repertoires, which serves as a basis for the list of BioInfoMiner calculation that, however, being of no relevance for the present study is not further developed. Finally, among the ranked proteins of interest, the profiles of peptide mappings from each CAF selection are compared by graphical presentation, which shows the amino acid sequence positions within the considered protein and the sum of the amino acids mappings hits (aah) by the peptides of the repertoire.

## 5. Conclusions

The application of PepSimili demonstrated that three individual peptide repertoires generated against a single cell line showed very high similarities, 57%, 61%, and 67% respectively, confirming the desired reproducibility of phage-displayed peptide selections. The mapping profiles of the peptides from the three selections to the human proteome reveal putative domains/epitopes that are mimicked by the selected peptides. Consequently, these mapping profiles are very similar among the most prominent mimicked proteins.

In perspective, the mapping of peptide selections to the whole proteome by the PepSimili tool provides major advantages. Methodologically, the PepSimili approach could comparatively evaluate NGS peptide selections of several first-round experiments performed in parallel, avoiding the enrichment biases caused in amplification rounds. The tool could be further extended in comparative selections with recombinant phage libraries encoding different sizes of peptides to define peptide ligands and mimicked protein domains/epitopes in biological studies, towards a very large spectrum of molecular targets both in vitro and in vivo.

## Figures and Tables

**Table 1:** Similarity evaluation of three CAF peptide selections at threshold 0.68. Given are the total numbers of peptides of the repertoires, and in bold the numbers of peptides in the peptide-to-peptide mapping between the repertoires using the PAM30 score; and the corresponding percentages of peptides in the repertoires with similarity at 0.68 threshold.

Similarity	CAF1	CAF2	CAF3
CAF1	616,009	**350,211** (57%)	**408,333** (66%)
CAF2		485,732	**378,635** (61%)
CAF3			578,315

**Table 2:** Lists of the first ranked proteins (named by gene symbol, BioInfoMiner) mapped by peptides of three CAF repertoires (PAM30 threshold 0.68). Color-highlighted proteins refer to examples retaken in Figure 4 showing the mapping profiles.

CAF1	CAF2	CAF3
PRIMA1	KRTAP4-16P	PRIMA1
FASLG	FASLG	KRTAP4-16P
WIPF3	RP5-1042K10.14	FASLG
ATF5	WIPF3	WIPF3
HOXB4	PRIMA1	C4orf46
KRTAP4-16P	ATF5	SMN2
MYPOP	C4orf46	ATF5
WASL	MYPOP	MYPOP
C4orf46	PRR11	HOXB4
WASF2	C4orf48	PRR11
NAF1	CTD-2545G14.7	NPIPB9
DMRTB1	HOXB4	RP5-1042K10.14
ACR	DMRTB1	NPIPB7
GJD3	ACR	ACR
RP5-1042K10.14	NCCRP1	NCCRP1
LMOD2	WASF2	DMRTB1
ZIC5	PRRG2	NPIPB8
NPIPB7	NPIPB7	PRRG2
PRR11	SMN2	WIPF1
SMN2	ZIC5	WASF2
NPIPB8	ANTXRL	SMN1
NPIPB9	WAS	NAF1
ANTXRL	NPIPB9	SMR3B
SMN1	NPIPB8	WASF1
NCCRP1	SF1	ZIC5
SMR3B	SMR3A	FMNL2
PRRG2	SMR3B	C19orf54
RAPH1	NAF1	SCX

## Data Availability

Publicly available datasets were analyzed in this study. Data are available in NIH Short Read Archive, PRJNA316731 (SAMN04589895-SAMN04589912).

## Supplementary Materials

The following supporting information can be downloaded at: https://www.mdpi.com/article/10.3390/ijms24021594/s1.
